# A humanized Gs-coupled DREADD for circuit and behavior modulation

**DOI:** 10.3389/fncel.2025.1577117

**Published:** 2025-04-09

**Authors:** Qi Zhang, Ruiqi Wang, Liang Zhang, Mengqi Li, Jianbang Lin, Xiaoyang Lu, Yixuan Tian, Yunping Lin, Taian Liu, Yefei Chen, Yuantao Li, Jun Cao, Qiang Wu, Jinhui Wang, Zhonghua Lu, Zexuan Hong

**Affiliations:** ^1^Department of Medicinal Chemistry and Natural Medicine Chemistry, College of Pharmacy, Harbin Medical University, Harbin, China; ^2^Research Center for Primate Neuromodulation and Neuroimaging, Institute of Biomedical and Health Engineering, Shenzhen Institute of Advanced Technology, Chinese Academy of Sciences, Shenzhen, China; ^3^Department of Anesthesiology, The Third People’s Hospital of Shenzhen, Shenzhen, China; ^4^Shenzhen Key Laboratory for Molecular Biology of Neural Development, Shenzhen Technological Research Center for Primate Translational Medicine, Shenzhen-Hong Kong Institute of Brain Science, Shenzhen Institute of Advanced Technology, Chinese Academy of Sciences, Shenzhen, China; ^5^University of Chinese Academy of Sciences, Beijing, China; ^6^Shenzhen Maternity and Child Healthcare Hospital, Southern Medical University, Shenzhen, China; ^7^State Key Laboratory of Biomedical Imaging Science and System, Key Laboratory of Biomedical Imaging Science and System, Chinese Academy of Sciences, Shenzhen, China

**Keywords:** DREADD, neuronal activation, modulation, transgene modification, Gs-signaling, D1-MSNs

## Abstract

Designer receptors exclusively activated by designer drugs (DREADDs) play important roles in neuroscience research and show great promise for future clinical interventions in neurological diseases. The Gs-coupled DREADD, rM3Ds, modulates excitability in neuron subsets that are sensitive to downstream effectors of Gs protein. However, given the non-human nature of the rM3Ds backbone, risks about potential immunogenicity and tolerability exist when considering clinical translation. Here, we report the development of a whole sequence-humanized Gs-coupled DREADD, hM3Ds. We found that hM3Ds has a comparable DREADD ligand response profile to rM3Ds. We then selectively expressed hM3Ds in D1 medium spiny neurons (D1-MSNs) and found that hM3Ds was able to activate the D1-MSNs-mediated basal ganglia direct pathway and alleviate Parkinsonian phenotypes in a Parkinson’s disease mouse model. In conclusion, this engineered humanized Gs-coupled DREADD is suitable as an effective, and likely safer, DREADD tool for both research and future clinical applications.

## 1 Introduction

Designer receptors exclusively activated by designer drugs (DREADDs) have been widely adopted in neuroscience research for activation or inhibition of DREADD-expressing cells (English and Roth, 2015; Roth, 2016). They play important roles in understanding the structural basis and functional characteristics of neural circuits. DREADDs are modified from different muscarinic acetylcholine receptors (M1R-M5R). By introducing two point mutations (Y3.33C and A5.46G) (Armbruster et al., 2007), DREADDs display extremely low sensitivity to the endogenous ligand acetylcholine, whilst it can be selectively activated by inert designer drugs. DREADDs can be classified according to the coupling G-proteins, such as Gq-coupled DREADDs (hM1Dq, hM3Dq, and hM5Dq), which activate Gq signaling through phospholipase-C and induce Ca^2+^ release (Hannan and Hall, 1993; Armbruster et al., 2007), and Gi-coupled DREADDs (hM2Di and hM4Di), which trigger Gi signaling by inhibiting adenylate cyclase and downstream cAMP production (Hannan and Hall, 1993; Armbruster et al., 2007). In addition, there is also the Gs-coupled DREADD (rM3Ds), which activates Gs/Golf signaling and increases intracellular cAMP concentrations (Guettier et al., 2009; Farrell et al., 2013), and the *β*-arrestin DREADD, which activates β-arrestin signaling (Nakajima and Wess, 2012).

Carried by adeno-associated virus (AAV) vectors, DREADDs can be delivered to brain regions of interest and maintain long-term expressions in target neurons (Pickering and Mazarakis, 2021; Buchlis et al., 2012). DREADDs actuators such as clozapine N-oxide (CNO), compound 21 (C21), deschloroclozapine (DCZ), and clozapine, can dose-dependently activate DREADDs (Nagai et al., 2020; Gomez et al., 2017; Atasoy and Sternson, 2018; Thompson et al., 2018) via different administration methods such as orally, via eye-drops, or via either intramuscular, intraperitoneal, or intravenous administration (Keenan et al., 2017; Zhan et al., 2019; Schalbetter et al., 2021; Oyama et al., 2022). Given its simplicity of use, reversibility and precise temporal control (Clark et al., 2024), DREADDs technology has been successfully applied in both rodents and non-human primates (Nagai et al., 2016; Roseboom et al., 2021; Galvan et al., 2019). More importantly, many studies have illustrated the ability of DREADDs to modulate neuronal activity and alleviate disease-associated phenotypes in several disorders such as amyotrophic lateral sclerosis (Saxena et al., 2013), Parkinson’s disease (PD) (Dell’Anno et al., 2014), epilepsy (Katzel et al., 2014; Krook-Magnuson and Soltesz, 2015), autism (Penagarikano et al., 2015), addiction-related disorders (Anderson et al., 2013; Ferguson et al., 2011), and Down’s syndrome (Fortress et al., 2015). These studies attest that DREADDs is a powerful technology and indicate great potential for future clinical applications in the treatment of neurological diseases.

The most commonly employed DREADD for activation is hM3Dq, which can upregulate activity in many neuronal and non-neuronal cell types (Armbruster et al., 2007; Alexander et al., 2009; Agulhon et al., 2013; Li et al., 2013). When combined with cell-specific promoters/enhancers, and/or other delivery systems with specific cell types or brain region tropisms (Lerchner et al., 2014), precise chemogenetic modulation in defined cell populations can be achieved. It is thought that the mechanism whereby ligand-induced Gq decouples from hM3Dq, leading to a rise in intracellular calcium concentration, is capable of enhancing excitability of most, if not all, neuronal subtypes after DREADD expression. In contrast, Gs-coupled rM3Ds can modulate neuronal excitability of more limited neuronal subtypes because only very limited types of neurons respond to Gs-coupled signaling events with a rise in excitability (Roth, 2016). In previous work, we showed that nigral injection of retrograde AAV containing rM3Ds, but not hM3Dq, exclusively activated D1-MSNs, without activating neurons near the injection sites in the substantia nigra upon systematic CNO administration (Chen et al., 2023). Specific activation of D1-MSNs-mediated the basal ganglia (BG) direct pathway and significantly reversed Parkinsonian symptoms in both PD mice and monkey models. Although rM3Ds shows great potential for therapeutic application, given that it is a non-human protein sequence (Guettier et al., 2009), concerns have emerged in terms of potential immunogenicity, long-term stability and efficiency (Sharon and Kamen, 2018; Bessis et al., 2004). Thus, a Gs-coupled DREADD engineered from a human muscarinic acetylcholine receptor would likely be safer and more stable in future clinical applications.

In this study, we modified rM3Ds into hM3Ds, a fully sequence-humanized Gs-coupled DREADDs. We compared the expression and the DREADD ligand response profile of hM3Ds and rM3Ds *in vitro*. Furthermore, we also evaluated its potential for chemogenetic activation of neurons *in vivo*. We selectively expressed hM3Ds in D1-MSNs and assessed the activation of the D1-MSNs-mediated direct pathway of the BG circuit and the related behavioral phenotype in PD mice.

## 2 Materials and methods

### 2.1 Sequence of engineered receptors

#### 2.1.1 hM3-ti23-Ds protein sequence

MTLHNNSTTSPLFPNISSSWIHSPSDAGLPPGTVTHFGSYNVSRAAGNFSSPDGTTDDPLGGHTVWQVVFIAFLTGILALVTIIGNILVIVSFKVNKQLKTVNNYFLLSLACADLIIGVISMNLFTTYIIMNRWALGNLACDLWLAIDCVASNASVMNLLVISFDRYFSITSPFRYQSLMTRARAGVMIGLAWVISFVLWAPAILFWQYFVGKRTVPPGECFIQFLSEPTITFGTAIAGFYMPVTIMTILYWRVYREAKEQIRKIDRCEGRFYGSQEQPQPPPLPQHQPILGNGRASKRKTSRVMAMREHKALQTLSAILLAFIITWTPYNIMVLVNTFCDSCIPKTFWNLGYWLCYINSTVNPVCYALCNKTFRTTFKMLLLCQCDKKKRRKQQYQQR QSVIFHKRAPEQAL*.

#### 2.1.2 hM3Ds protein sequence

MTLHNNSTTSPLFPNISSSWIHSPSDAGLPPGTVTHFGSYNVSRAAGNFSSPDGTTDDPLGGHTVWQVVFIAFLTGILALVTIIGNILVIVSFKVNKQLKTVNNYFLLSLACADLIIGVISMNLFTTYIIMNRWALGNLACDLWLAIDCVASNASVMNLLVISFDRYFSITSPFRYQSLLTRARAGVMIGLAWVISFVLWAPAILFWQYFVGKRTVPPGECFIQFLSEPTITFGTAIAGFYMPVTIMTILYWRVFREAQKQVKKIDSCERRFLGGPARPPSPSPSPVPAPAPPPGPPRPAAAAATAPLANGRAGKRRPSRLVALREQKALQTLSAILLAFIITWTPYNIMVLVNTFCDSCIPKTFWNLGYWLCYINSTVNPVCYALCNKTFRTTFKMLLLCQCDKKKRRKQQYQQRQSVIFHKRAPEQAL*.

### 2.2 Vector construction

Sequences of *hM3-ti23-Ds* and *hM3Ds* were synthesized by GENEWIZ Co., Ltd. To generate *pAAV-CMV-hM3-ti23-Ds, hM3-ti23-Ds* was subcloned into *pAAV-CMV-EYFP* to replace *EYFP* and to generate *pAAV-CMV-hM3Ds, hM3Ds* was subcloned into *pAAV-CMV-EYFP* to replace *EYFP.* To generate *pAAV-CMV-rM3Ds*, *rM3Ds* was subcloned from *pAAV-G88P7-rM3Ds-2A-EYFP* (Addgene, 213970) into *pAAV-CMV-EYFP* to replace *EYFP* by restriction enzyme digestion. To generate *pAAV-CMV-hM3Dq*, *hM3Dq* was subcloned from *pAAV-G88P3-HA-hM3Dq* (Addgene, 213972) into *pAAV-CMV-EYFP* to replace *EYFP.* To generate *pAAV-CMV-HA-hM3-ti23-Ds/hM3Ds/rM3Ds*, a HA-tag fragment was introduced into *pAAV-CMV-hM3-ti23-Ds/hM3Ds/rM3Ds* using primers containing HA-tag sequence. M1-M10 mutations were introduced into *pAAV-CMV-hM3Ds* using primers containing mutated amino acids. To generate *pAAV-G88P7-hM3Ds-2A-EYFP*, hM3Ds was subcloned into *pAAV-G88P7-rM3Ds-2A-EYFP* via restriction enzyme digestion. Constructed plasmids were subjected to Sanger sequencing for sequence confirmation.

### 2.3 AAV packaging

For packaging of the AAV8R12-*G88P7-hM3Ds-2A-EYFP* vector, the AAV8R12 Rep-Cap plasmid (Addgene, 213968), the *pAAV-G88P7-hM3Ds-2A-EYFP* plasmid and the *pAdDeltaF6* helper plasmid (Addgene, 112867) were co-transfected into HEK293T cells with calcium phosphate. Cells were harvested 72 h after transfection and resuspended in resuspension buffer (150 mM NaCl, 100 mM Tris–HCl, pH 8.0). The virus particles were released using consecutive freeze/thaw cycling and then purified and concentrated using ultrafiltration tubes (100KD, Merck Millipore). The viral titer was analyzed by extraction of viral DNA and subsequent quantitative polymerase chain reaction analysis using primers specific to the viral ITR sequences. The obtained virus particles were denatured with Proteinase K and stored at −80°C for use.

### 2.4 Cell transfection

For the detection of protein expression, HEK293T cells (ATCC) were seeded in 6-well plate with Dulbecco’s modified Eagle’s medium (DMEM) containing 10% fetal bovine serum (Gibco). Upon reaching ~80% confluency, cells were transfected with 3 μg HA-tagged engineered receptors or HA-tagged rM3Ds plasmids (jetOPTIMUS^®^ transfection reagent). The medium was changed to fresh complete medium 24 h after transfection.

As for the cAMP assay, HEK293T cells (ATCC) were seeded in 6-well plate with DMEM containing 10% fetal bovine serum. Upon reaching ~80% confluency, cells were transfected with DREADDs plasmids (30 ng), *pCRE-Luc* (GeneBank accession: AF053461.1, 600 ng) and *pRL-SV40* (GeneBank accession: AF025845.2, 150 ng). The following day, cells were redistributed from 6-well plates to white opaque 96-well plates pre-coated with poly-L-lysine (Solarbio).

As for the Ca^2+^ imaging assay, HEK293T cells (ATCC) were seeded in 24-well plate with DMEM containing 10% fetal bovine serum. Upon reaching ~80% confluency, cells were cotransfected with *pAAV-CAG-GCaMP6s* (0.1 μg‌), and *pAAV-CMV-hM3Ds* or *pAAV-CMV-hM3Dq* (0.1 μg).

### 2.5 cAMP assay

After 24 h of seeding in 96-well plates, the medium in each well was replaced with 100 μL CNO (Hello Bio) or 100 μL acetylcholine chloride (MCE) solution, which were diluted with complete medium to different concentrations. The plates were then placed back in the cell incubator for 6 h. The cAMP level was detected using a Dual Glo Luciferase Reporter Gene Assay Kit (YEASEN) following the manufacturer’s protocol. The data of each well was presented as the ratio of firefly luciferase luminescence to renilla luciferase luminescence.

### 2.6 Ca^2+^ imaging

48 h after transfection, the medium in each well was replaced with 400 μL CNO (10 μM) solution, or with 400 μL vehicle (medium only). The plates were then placed back in the cell incubator for 10 min. Cell fluorescence were imaged under microscopy and quantified using ImageJ.

### 2.7 Western blot

Cells were lysed with RIPA buffer 72 h after cell transfection to extract protein. After denaturation, the protein samples were separated on a 4–12% sodium dodecyl sulfate polyacrylamide gel (YEASEN) and subsequently transferred to polyvinylidene fluoride membranes (Millipore). The membranes were blocked at room temperature for 1 h and then incubated with anti-HA antibody (BioLegend, 923501) or anti-GAPDH (Abcam, ab70699), diluted to 1:1000 overnight at 4°C. The following day, the membranes were immersed with anti-rabbit or anti-mouse secondary antibodies conjugated with horseradish peroxidase (APExBIO, K1223 and K1221) at room temperature for 1 h. Immunoreactive bands were detected using a hypersensitive ECL Chemiluminescent Substrate Detection Kit (APExBIO). Grayscale analysis of protein bands was conducted using ImageJ.

### 2.8 Animals

Male C57BL/6 J mice (8–week’s old) were housed under pathogen-free conditions with free access to food and water. All experiments were approved by the Institutional Animal Care and Use Committee (IACUC) at Shenzhen Institute of Advanced Technology, Chinese Academy of Sciences, following the guidelines stated in the Guide for Care and Use of Laboratory Animals (Eighth Edition, 2011).

### 2.9 Stereotactic injection

#### 2.9.1 AAV injection

Animals were anesthetized via intraperitoneal injection of pentobarbital sodium (Sigma) and placed into a stereotactic frame (RWD Instruments). An incision was made to expose the skull and locate the site above the SNr (from bregma: AP –3.4 mm, ML 1.3 mm, DV –4.8 mm). A hole in the skull was drilled above the SNr using an electrodrill (RWD Instruments). The AAV8R12-*G88P7-hM3Ds-2A-EYFP* vector (200 nL volume) was injected using a 10 μL 33-Gauge Hamilton syringe at a rate of 20 nL/min. The syringe was withdrawn 10 min after finishing injection. Behavioral experiments are performed at least 3 weeks after virus injection.

#### 2.9.2 6-hydroxydopamine (6-OHDA) injection

A PD mouse model was generated using 6-OHDA. Desipramine (Sigma) was administrated (25 mg/kg) via intraperitoneal injection 30 min before a 6-OHDA injection. The stereotactic injection procedure was identical to the AAV injection described above. A volume of 800 nL 6-OHDA (5 mg/mL) was injected into each side of striatum (from bregma: AP 0.5 mm, ML ±1.5 mm, DV –3.2 mm) at a rate of 100 nL/min. One week after 6-OHDA lesions were generated, AAV injections were administered, and behavioral tests were conducted 3 weeks after AAV injections.

### 2.10 Behavior measurements

#### 2.10.1 Open field test

Mice were tested in an open field box arena with length, width and height of 50 × 50 × 50 cm. The animals underwent 3 days of habituation to the environment and experimenter and 1 day of habituation to the test apparatus. On the test day, the animals were injected with saline or CNO (0.3 mg/kg). The animals were placed into the test box 30 min after injection and video was recorded for 10 min. The number of rotations, distance traveled, and time spent immobile were then quantified using Anymaze software and the video recordings.

#### 2.10.2 Rotarod test

After 3 days of habituation to the environment and operator, the mice were placed on a suspended rod apparatus (Xinruan) and trained at speeds of 10, 20, 30 and 40 rpm/min for 2 days. Each training session used one speed and lasted 10 min; a 15 min interval was then given before moving on to the next speed. Each mouse was trained at all speeds each day. On the test day, each mouse underwent three trials (10, 20, 30 and 40 rpm/min, test duration: 10 min) with a resting time of 15 min between each trial. The mean latency to fall was used for further calculations.

### 2.11 Immunofluorescence staining

Animals were deeply anesthetized and perfused with saline and 4% paraformaldehyde perfusion through left ventricle. The mouse brains were extracted, fixed overnight, and then dehydrated with 30% sucrose solution. After being embedded in OCT (Sakura), brain sections were prepared at 40 μm thickness by a cryostat (Leica, CM1950).

Brain slices were blocked and permeabilized with PBS containing 5% bovine serum albumin (BSA) and 0.3% Triton X-100 for 30 min. The slices were then incubated with anti-GFP (Abcam, ab13970) or anti-TH (Abcam, ab76442), diluted to 1:500 in PBS containing 5% BSA at 4°C overnight. The sections were then incubated with diluted Alexa Fluor 488 goat anti-chicken IgG antibody (Thermo Fisher, A-11039) or Alexa Fluor 594 donkey anti-mouse IgG antibody (Thermo Fisher, A32744) at room temperature for 1 h. DAPI (Sigma, D9542) was also applied to stain the nuclei. Images were acquired using a slide scanner (Olympus, BX61VS) and a confocal microscope (Zeiss, LSM880).

### 2.12 Statistical analysis

The data are expressed as the mean ± standard error and analyzed using GraphPad Prism 9.0. Paired *t*-test, unpaired *t*-test, one-way ANOVA, Tukey’s test, and Dunnett’s test were used where appropriate. All *t*-tests were performed as two-tailed. A *p*-value <0.05 was considered statistically significant. All statistical tests used are indicated in the figure legends.

## 3 Results

### 3.1 Generation of a humanized Gs-coupled hM3 receptor

The Gs-coupled DREADD, rM3Ds, is composed of a mutant rat M3R receptor (Y148C and A238G point mutations) with the second and third intracellular loop (i2 and i3) replaced with the corresponding sequence of Gs-coupled turkey erythrocyte β1-adrenergic receptor (β1-AR) (Guettier et al., 2009).

**Figure 1:**
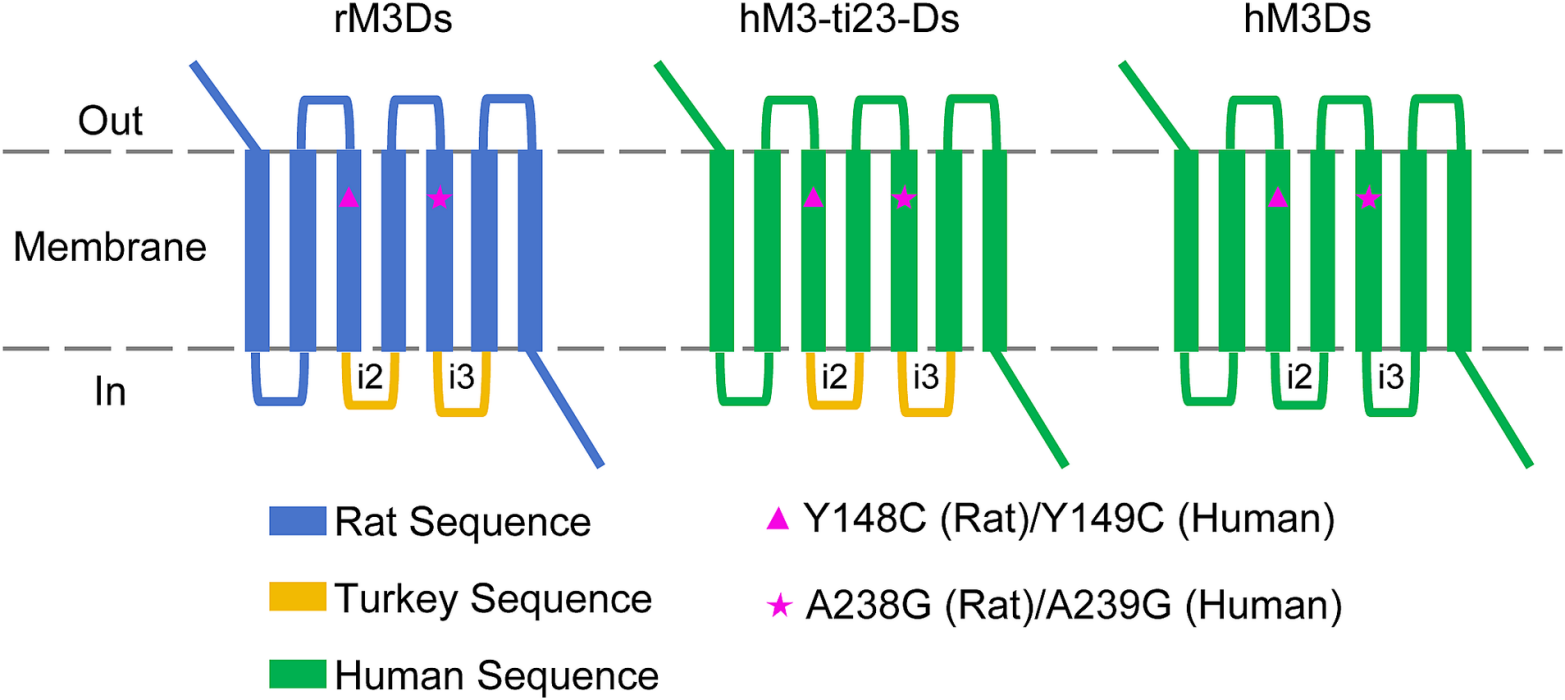
Schematic diagram of the engineered humanized Gs-coupled DREADD. The rat M3R sequence from rM3Ds was replaced with hM3R followed by corresponding Y149C and A239G point mutations (hM3R sequence). The i2 and i3 region of turkey β1-AR were also replaced with i2 and i3 sequences of human β1-AR, respectively.

Similarly, we first swapped the rat M3R with the corresponding sequence of human M3R and retained the i2 and i3 loop of turkey β1-AR. In addition, the Y149C and A239G point mutations of human M3R (corresponding to Y148C and A238G of the rat M3R) were introduced, which had the effect of switching the receptor to respond to CNO or DCZ instead of the endogenous ligand, acetylcholine (ACh) (Armbruster et al., 2007). This mutant receptor is referred to as hM3-ti23-Ds (human M3 muscarinic with turkey i2&3 DREADD receptor coupled to Gs) (Figure 1; Supplementary Figure S1).

To obtain full sequence-humanized Gs-coupled DREADD, we then replaced the i2 and i3 loop containing turkey β1-AR with human β1-AR. This humanized mutant receptor is referred to as hM3Ds (human M3 muscarinic DREADD receptor coupled to Gs) (Figure 1; Supplementary Figure S1).

### 3.2 Ligand binding and G-protein binding properties of mutant DREADDs

**Figure 2:**
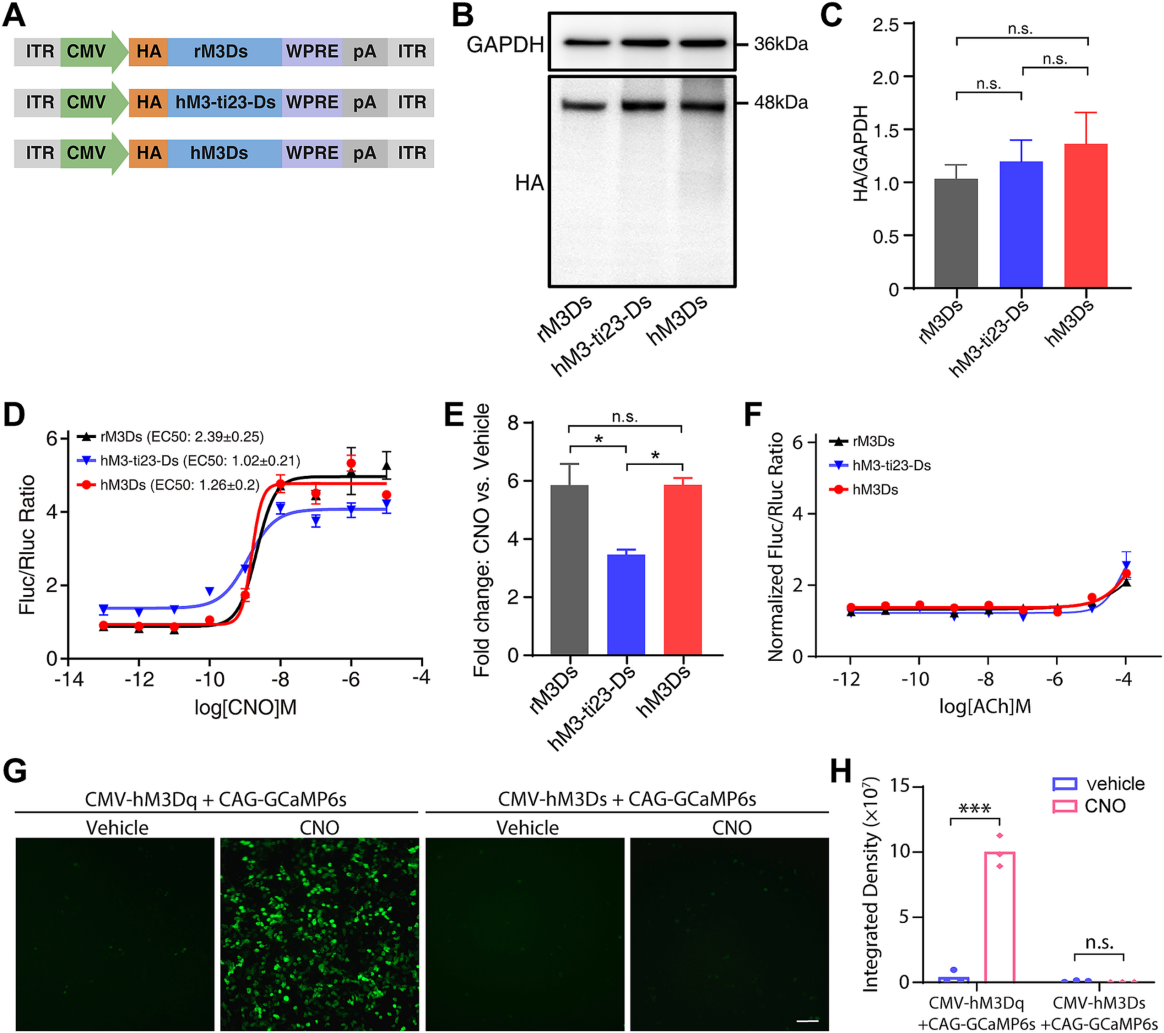
Responses of engineered Gs receptors to CNO administration *in vitro*. **(A)** Schematic showing the HA-tagged rM3Ds, hM3-ti23-Ds and hM3Ds. ITR, inverted terminal repeat; WPRE, woodchuck hepatitis virus post-transcriptional regulatory element; pA, polyadenylation signal. **(B)** Western blot showing expression levels of rM3Ds, hM3-ti23-Ds and hM3Ds in HEK293T cells. **(C)** Comparison between expression levels of rM3Ds, hM3-ti23-Ds and hM3Ds. GAPDH was used as a loading control. Data are shown as mean ± SEM of triplicate experiments, one-way ANOVA with Tukey’s post-hoc test, n.s., not significant. **(D)** Luciferase reporter assay after CNO administration in HEK293T cells expressing with rM3Ds, hM3-ti23-Ds or hM3Ds. cAMP levels are indicated as ratios of firefly to renilla luciferase activity. **(E)** Luminescence fold changes elicited by rM3Ds, hM3-ti23-Ds and hM3Ds with excessive concentration CNO at 10-μM concentration. Data are shown as mean ± SEM of triplicate experiments, one-way ANOVA with Tukey’s post-hoc test, ** *p* < 0.01, n.s., not significant. **(F)** Luciferase reporter assay after ACh administration in HEK293T cells expressing with rM3Ds, hM3-ti23-Ds or hM3Ds. cAMP levels are indicated as normalized ratios of firefly to renilla luciferase activity. **(G,H)** Representative calcium image **(G)** and fluorescence quantification **(H)** of hM3Ds- and hM3Dq- transfected cells after vehicle or CNO treatment. Scale bar, 50 μm. Data are represented as mean ± SEM, two-tailed unpaired *t* test, *** *p* ≤ 0.001, n.s., not significant.

To investigate the protein expression of hM3-ti23-Ds and hM3Ds and to compare this with rM3Ds, we added an N-terminal HA tag to the N-terminus of these receptors (Figure 2A). After transfection in HEK293T cells, western blots revealed that hM3-ti23-Ds and hM3Ds had a similar expression level to that of rM3Ds (Figures 2B,C).

We next examined whether the chemogenetic receptor ligand, CNO, could stimulate hM3-ti23-Ds and hM3Ds and induce up-regulation of cAMP level. We co-transfected HEK293T cells with engineered receptors and the reporter vector, pCRE-Luc, which contains a cAMP response element-driven firefly luciferase (Luc) (Bokar et al., 1988); pRL-SV40 encoding renilla luciferase was simultaneously transfected as a transfection efficiency control.

A luciferase assay revealed that CNO treatment dose-dependently increased the cAMP response with either hM3-ti23-Ds or hM3Ds (Figure 2D). We found that the baseline level of Luc activity in hM3-ti23-Ds-transfected cells was higher than the baselines of hM3Ds- and rM3Ds-transfected cells, indicating a partial response of hM3-ti23-Ds to endogenous ligands or a relatively higher ligand-independent basal activity (Figure 2D). Surprisingly, the full-humanized engineered receptor, hM3Ds, had a similar response to CNO with a lower EC50 (EC50: 1.26 ± 0.2 nM) compared to rM3Ds (EC50: 2.39 ± 0.25 nM, Figure 2D). We also calculated the fold change in luciferase activity following CNO at 10 μM concentration and found a comparable fold change in luciferase elicited by hM3Ds to that elicited by rM3Ds, whilst hM3-ti23-Ds induced a lower fold change (Figure 2E).

We further evaluated the response of hM3-ti23-Ds or hM3Ds to ACh and found a modest elevation in luciferase activity following exposure to 100 μM of ACh in both constructs, along with rM3Ds-transfected cells (Figure 2F). This observation aligns with previous finding demonstrating ACh responsiveness in rM3Ds at high concentration (Armbruster et al., 2007). Besides, calcium assay showed that CNO administration did not induce detectable levels of calcium rise in hM3Ds-transfected cells (Figures 2G,H). Collectively, these results demonstrated that hM3Ds specifically activated Gs-mediated signaling pathways without activating Gq-related calcium responses.

Muscarinic acetylcholine receptors are expressed in many mammalian species and there are minor sequence discrepancies which might have contributed to their adaptation to various environments during evolution (Collin et al., 2013; Strotmann et al., 2011). We speculated that these sequence differences may change receptor characteristics in downstream signaling. Thus, we compared M3R amino acid sequences of ten species including humans, macaques, lemurs, bats, bears, pipistrellus, camels, meles, mirounga, leopards, marmotas, and rats (Supplementary Figure S2). We selected ten non-conserved amino acid residues and introduced corresponding point mutations into hM3Ds (Table 1). Considering that immunogenic epitopes typically comprise more than 7 amino acids (Kril et al., 2024; Rammensee et al., 1999) and these ten mutation sites are located outside the known peptidic epitope sequences documented in the UniProt database, we reasoned that these mutations would not significantly impact the immunogenicity profile of hM3Ds. We then determined whether these mutations can affect the response and potency of hM3Ds in HEK293T cells. We calculated the fold changes of luciferase activity following CNO at 10 μM concentration (Supplementary Figure S3A), and unfortunately, we did not find any mutants that outperformed hM3Ds. Among these mutant receptors, M397T (M1), K410R (M7), K423R (M8), A429D (M9) and L430S (M10) mutations elicited similar fold changes in luciferase as hM3Ds, whilst Q402R (M2), D404G (M3), K405R (M4), K406R (M5) and K407R (M6) mutations resulted in reduced luciferase activity compared to hM3Ds (Supplementary Figure S3B). Although we did not identify a mutant with better performance compared with the original hM3Ds DREADD, these results expand our knowledge on how sequence diversity might affect Gs-coupled signaling.

**Table 1:** Summary of point mutations of hM3Ds mutants.

Receptor mutants	Amino acid mutations
hM3Ds-M1	M397T
hM3Ds-M2	Q402R
hM3Ds-M3	D404G
hM3Ds-M4	K405R
hM3Ds-M5	K406R
hM3Ds-M6	K407R
hM3Ds-M7	K410R
hM3Ds-M8	K423R
hM3Ds-M9	A429D
hM3Ds-M10	L430S

### 3.3 hM3Ds efficiently activates the BG direct pathway and drives robust behavior change following CNO *in vivo*

**Figure 3:**
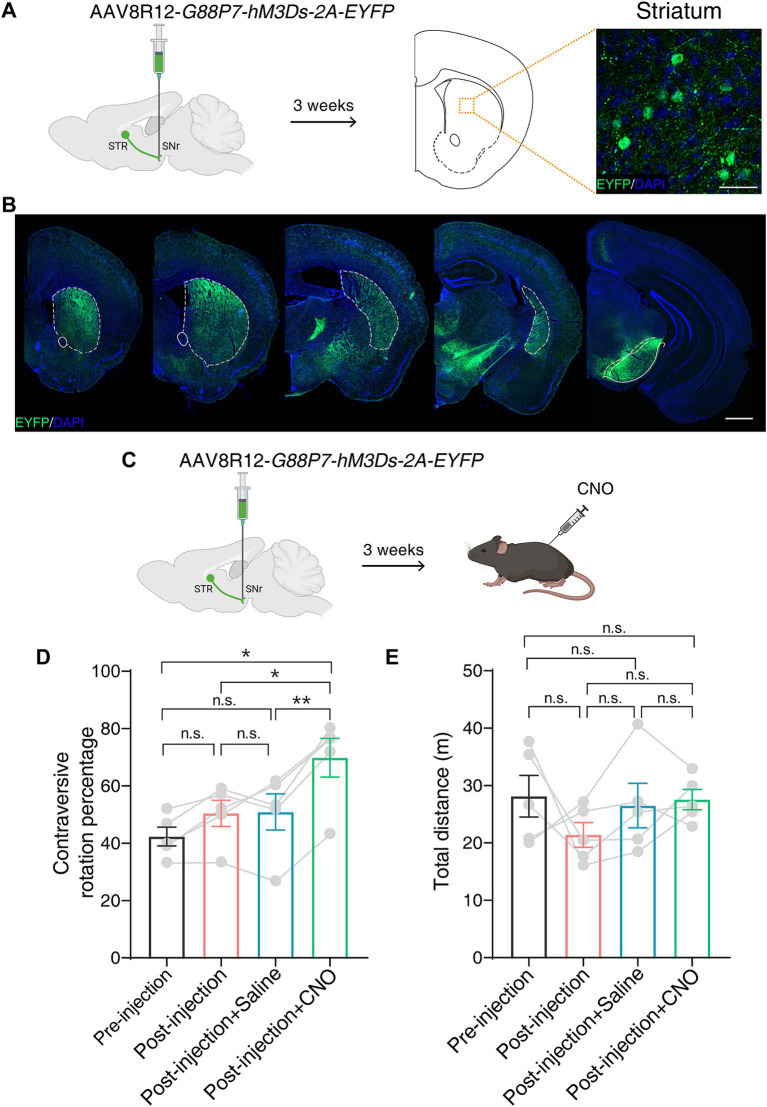
hM3Ds efficiently activates the BG direct pathway and drives robust behavior following CNO in mice. **(A)** Schematic showing unilateral AAV8R12-*G88P7-hM3Ds-2A-EYFP* injection into the SNr in mice (left) and a representative image showing labeled neurons (EYFP+) in the striatum (right). SNr, substantia nigra pars reticulate; STR, striatum. Scale bar, 20 μm. **(B)** Coronal whole-brain labeling pattern of labeled neurons (EYFP+) after SNr injection of AAV8R12-*G88P7-hM3Ds-2A-EYFP*. Dashed lines mark the striatum. Scale bar, 1 mm. **(C)** Schematic showing chemogenetic activation of the BG direct pathway following AAV8R12-*G88P7-hM3Ds-2A-EYFP* injection and intraperitoneal injection of CNO. **(D,E)** Calculation of contraversive rotation percentage **(D)** and quantification of distance traveled **(E)** in an open field test (*n* = 5 mice per group). Data are represented as mean ± SEM, one-way ANOVA with Tukey’s post-hoc test, * *p* < 0.05, ** *p* < 0.01, n.s., not significant.

To functionally validate hM3Ds *in vivo*, we adopted a previously developed AAV tool kit, which enables efficient and specific retrograde labeling of striatal D1-MSNs that are activated by cAMP level upregulation (Farrell et al., 2013; Chen et al., 2023). This AAV tool kit is able to modulate D1-MSNs activity using a chemogenetic approach in both mice and monkeys. We first unilaterally injected AAV8R12-*G88P7-hM3Ds-2A-EYFP* into the substantia nigra pars reticulate (SNr) in mice. Immunofluorescence revealed a labeling pattern limited to the striatum with a robust labeling of neurons in the striatum (Figures 3A,B).

The D1-MSNs-mediated direct BG pathway plays an important role in motor control (Kreitzer and Malenka, 2008). We performed an open field test 3 weeks after unilateral AAV delivery to the SNr (Figure 3C). We observed more contralateral rotation following intraperitoneal injection of CNO than following injection of saline, and no difference in the total distance traveled (Figures 3D,E). Taken together, these results indicate that hM3Ds is able to chemogenetically activate target neurons and drive robust motor behavior in mice.

### 3.4 Chemogenetic activation of the BG direct pathway via hM3Ds alleviated Parkinsonian symptoms in PD mice

DREADDs technology has great potential for clinical applications and PD is a good candidate for DREADD-based therapeutics (Roseboom et al., 2021; English and Roth, 2015; Urban and Roth, 2015). In PD, the activity of the direct BG pathway is underactive whilst the indirect pathway is overactive, resulting in motor defects including bradykinesia, resting tremor, rigidity, and postural disturbance (Poewe et al., 2017). In addition, it has been shown that chemogenetic activation of D1-MSNs mediates the direct pathway and rescues PD motor symptoms in both rodents and a non-human primate PD model (Alcacer et al., 2017; Chen et al., 2023). Given this, we wondered whether engineered hM3Ds could restore the BG direct pathway function and thus alleviate motor disorders in the PD mouse model.

**Figure 4:**
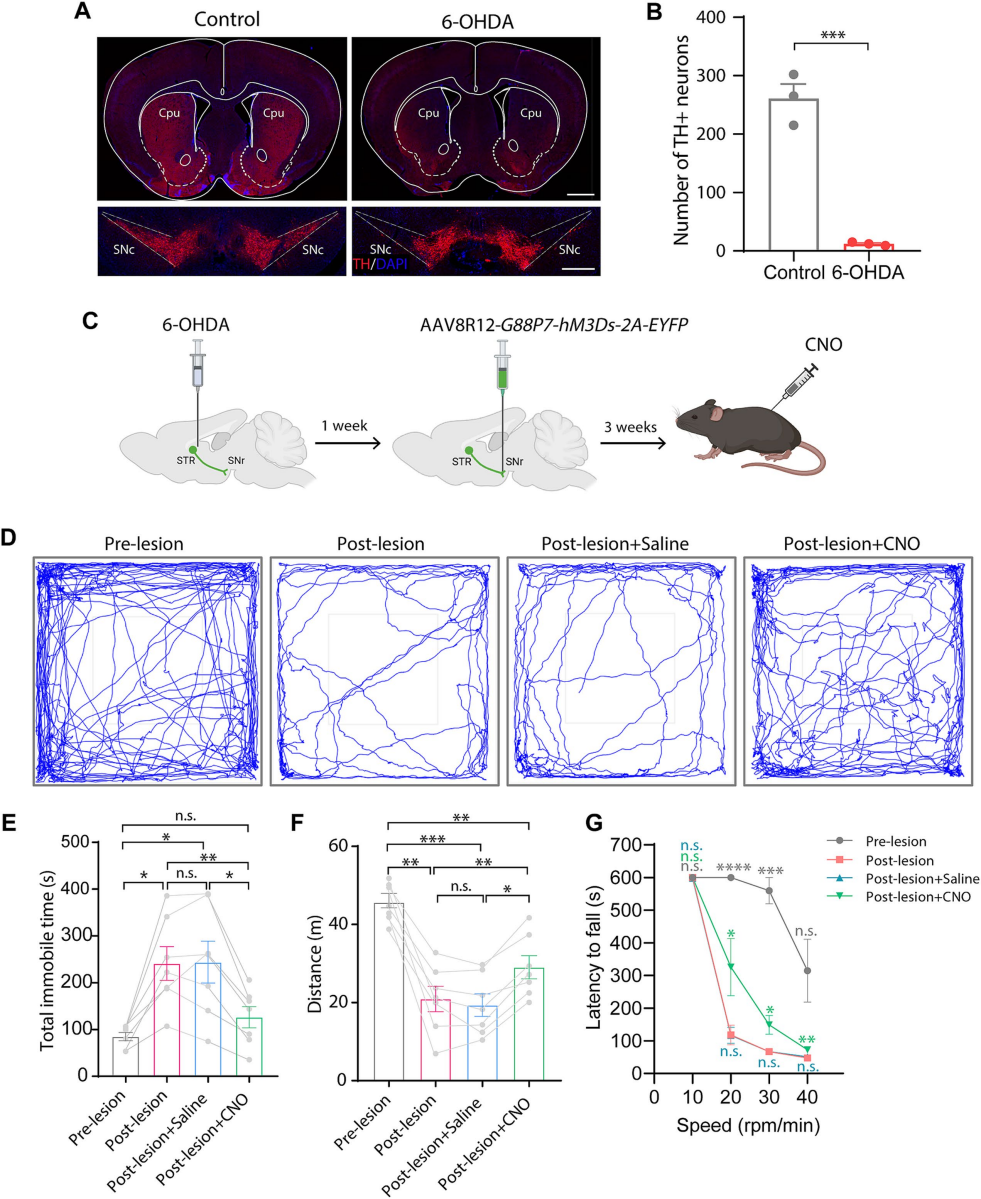
Chemogenetic activation of the direct pathway via hM3Ds alleviates behavioral movement deficits in PD mice. **(A)** TH-staining of the striatum and the SNc 1 week after bilateral 6-OHDA injection into the striatum. SNc, substantia nigra pars compacta. Scale bars, 1 mm (top) and 500 μm (bottom). **(B)** Dopaminergic neuron (TH+) count in the SNc (*n* = 3 mice per group). Data are represented as mean ± SEM, two-tailed unpaired *t* test, *** *p* < 0.001. **(C)** Experimental schematic showing chemogenetic activation of the BG direct pathway via 6-OHDA injection and nigral AAV8R12-*G88P7-hM3Ds-2A-EYFP* delivery, followed by CNO treatment. **(D)** Representative trajectory diagrams of mice in an open field test before and after PD modeling and after saline or CNO treatment in lesioned mice. **(E–G)** Quantification of time spent immobile **(E)** and total spontaneous motion distance traveled **(F)** in an open field test and latency to fall off the rotating rod **(G)** (*n* = 7 mice per group). Data are represented as mean ± SEM, one-way ANOVA with Tukey’s post-hoc test **(E,F)** and one-way ANOVA with Dunnett’s post-hoc test **(G)**, * *p* < 0.05, ** *p* < 0.01, *** *p* < 0.001, **** *p* < 0.0001, n.s., not significant.

To test this, we bilaterally injected 6-OHDA into the striatum to create a PD model, which led to an almost complete loss of both dopaminergic neurons in the SNc and dopaminergic innervation in the striatum (Figures 4A,B). We then bilaterally injected AAV8R12-*G88P7-hM3Ds-2A-EYFP* into the SNr. Three weeks after AAV delivery, we conducted open-field and rotarod tests (Figure 4C). We found that mean distance traveled was lower and time spent immobile was higher after 6-OHDA modeling, both of which were reversed upon CNO administration (Figures 4D–F). Furthermore, rotarod tests showed that the latency to fall was markedly shorter in PD mice than in before 6-OHDA lesion test and that there was partial recovery of motor skills following CNO treatment (Figure 4G). These data confirm hM3Ds’s effectiveness in restoring circuit function and reversing PD behavioral phenotypes, and more importantly, showcase its therapeutic utility in PD.

## 4 Discussion

In this study, we report a new Gs-coupled humanized DREADD, hM3Ds, which is derived from human M3R and human β1-AR. Engineered hM3Ds exhibits comparable ligand binding properties and Gs-signaling activation to rM3Ds. We also used hM3Ds to activate striatal D1-MSNs and to relieve motor defects in PD mice. Our results suggest that hM3Ds can be employed as a preferred chemogenetic tool with high clinical translational potential.

There is much current research investigating DREADDs-based therapeutics for a wide range of neurological disorders. In general, DREADDs are delivered by AAV, the leading vector choice for gene therapy. One major challenge for gene therapy is secondary immune response that affects transgene persistence and therapeutic safety. Numerous studies have demonstrated host immunogenicity to the AAV capsid and transgene product in clinical trials (Bessis et al., 2004; Mingozzi and High, 2017). Strategies to overcome this issue include the use of immunosuppressant drugs, switching AAV serotype, reducing vector dosage, and plasmapheresis (Monteilhet et al., 2011; Mingozzi et al., 2012; Wang et al., 2019). CpG depletion of the AAV vector genome (Faust et al., 2013) and incorporation of a toll-like receptor 9-inhibitory DNA sequence into the rAAV genome (Chan et al., 2021) are also promising approaches for immune modulation. Yet, these methods, some of which have been tested clinically, mainly tackle immunogenicity to AAV capsids and vector backbone genome. Immune response to transgene products remains a long-term issue since transgenes persistently encode proteins that may trigger B-cell-mediated and T-cell-mediated adaptive responses (Mendell et al., 2010; Wang et al., 2019). Thus, transgene modifications are also vital to suppress the immune response. The most common currently used Gs-coupled DREADD, rM3Ds, is composed of rat M3R and turkey β1-AR (Guettier et al., 2009). The sequence homology between rat M3R and human M3R is high, whilst the sequence homology of i2&i3 between turkey β1-AR and human β1-AR is low. Transgene codon optimization, especially for species-specific protein, is important to maintain sufficient protein expression and regulate immune responses (Mingozzi and High, 2017; Li and Samulski, 2020). Thus, we assume that sequence-humanized hM3Ds is more feasible than rM3Ds for successful clinical application.

In the present study, we validated the ability of hM3Ds to chemogenetically activate D1-MSNs, which can be activated by cAMP. Another G-protein subunit, Golf, is highly homologous to Gs and also promotes cAMP production (Yano et al., 2017). Thus, hM3Ds is likely applicable in other cell populations that use cAMP as a second messenger through Gs/Golf-signaling. In the central nervous system, Gs is mainly expressed in the cortex (Herve, 2011), whereas Golf expression is widespread across the whole brain, particularly in the striatum and olfactory tubercle (Zhuang et al., 2000; Millett et al., 2024). According to our results, hM3Ds can trigger cAMP accumulation, which indicates possible utility of hM3Ds in cell types in the brain that express Gs/Golf.

The main aim of our study was to develop a humanized Gs-coupled DREADD for neuronal modulation as a candidate for clinical translation. Although point mutations (Y149C and A239G) in hM3R DREADDs have been shown to have minor sensitivity to ACh (Armbruster et al., 2007), which was also validated in our study. Their response to the endogenous ligand of hM3Ds needs further confirmation. Another challenge to the translational potential of DREADDs is that the currently used ligands (CNO or DCZ) are not Food and Drug Administration-approved drugs. It has been proposed that clinically approved drugs such as clozapine or olanzapine be adopted as potential agonists for DREADDs (Weston et al., 2019; Jendryka et al., 2019). Thus, more research is needed to clarify the ligand binding and response characteristic of hM3Ds. Overall, we posit that hM3Ds can serve as an effective and safe DREADD tool for future preclinical and clinical applications.

## Data Availability

The original contributions presented in the study are included in the article/Supplementary material, further inquiries can be directed to the corresponding authors.

## Supplementary material

SUPPLEMENTARY FIGURE S1 Amino acid sequence alignment of rM3Ds, hM3-ti23-Ds and hM3Ds. Amino acid sequence alignments of rM3Ds, hM3-ti23-Ds and hM3Ds. Orange triangles show Y148C (rat)/Y149C (human) and A238G (rat)/A239G (human) mutations. i2 and i3 loops of DREADDs are highlighted in orange boxes.

SUPPLEMENTARY FIGURE S2 Amino acid sequence alignments of M3R of humans, macaques, lemurs, bats, bears, pipistrellus, camels, meles, mirounga, leopards, marmotas, and rats. The bottom panel is an enlarged image showing the point mutations of hM3Ds-M1-M10 (red arrowhead).

SUPPLEMENTARY FIGURE S3 Response of engineered hM3Ds mutants to CNO. **(A)** Experimental diagram showing assessment of the response of hM3Ds mutants to CNO, compared with rM3Ds and hM3Ds. **(B)** Relative luminescence fold changes elicited by rM3Ds, hM3Ds and hM3Ds mutants with excessive concentration of CNO at 10 μM. Data are normalized to the fold change elicited by hM3Ds and shown as mean ± SEM of triplicate experiments, one-way ANOVA with Dunnett’s post-hoc test, * *p* < 0.05, ** *p* < 0.01, n.s., not significant.
