# Supplementary figures and images for: A humanized Gs-coupled DREADD for circuit and behavior modulation
Source: Front Cell Neurosci. 2025 Apr 9;19:1577117. doi: 10.3389/fncel.2025.1577117 (PMC12015759; doi:10.3389/fncel.2025.1577117)

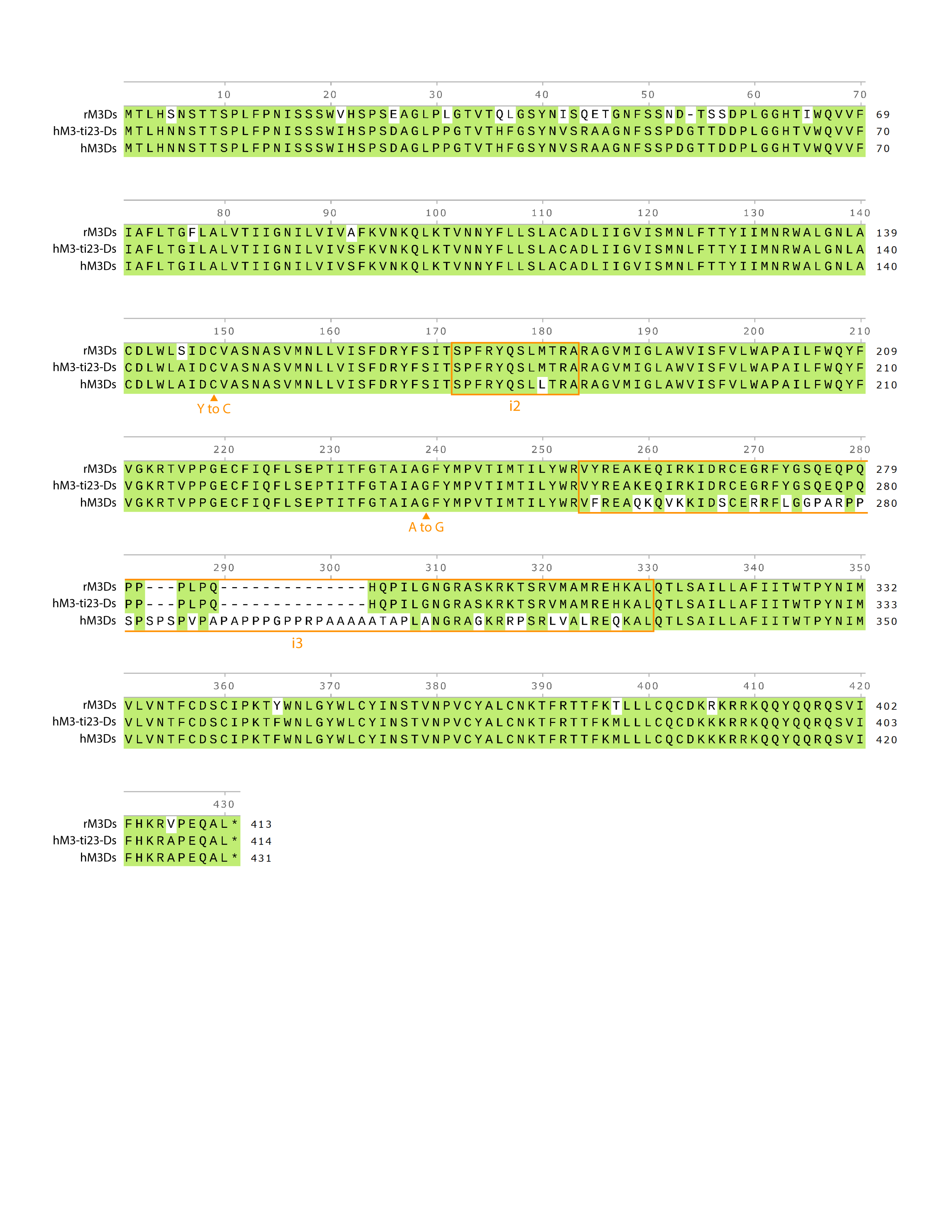

Supplement: SUPPLEMENTARY FIGURE S1 — Amino acid sequence alignment of rM3Ds, hM3-ti23-Ds and hM3Ds. Amino acid sequence alignments of rM3Ds, hM3-ti23-Ds and hM3Ds. Orange triangles show Y148C (rat)/Y149C (human) and A238G (rat)/A239G (human) mutations. i2 and i3 loops of DREADDs are highlighted in orange boxes. [file Image_1.TIF]

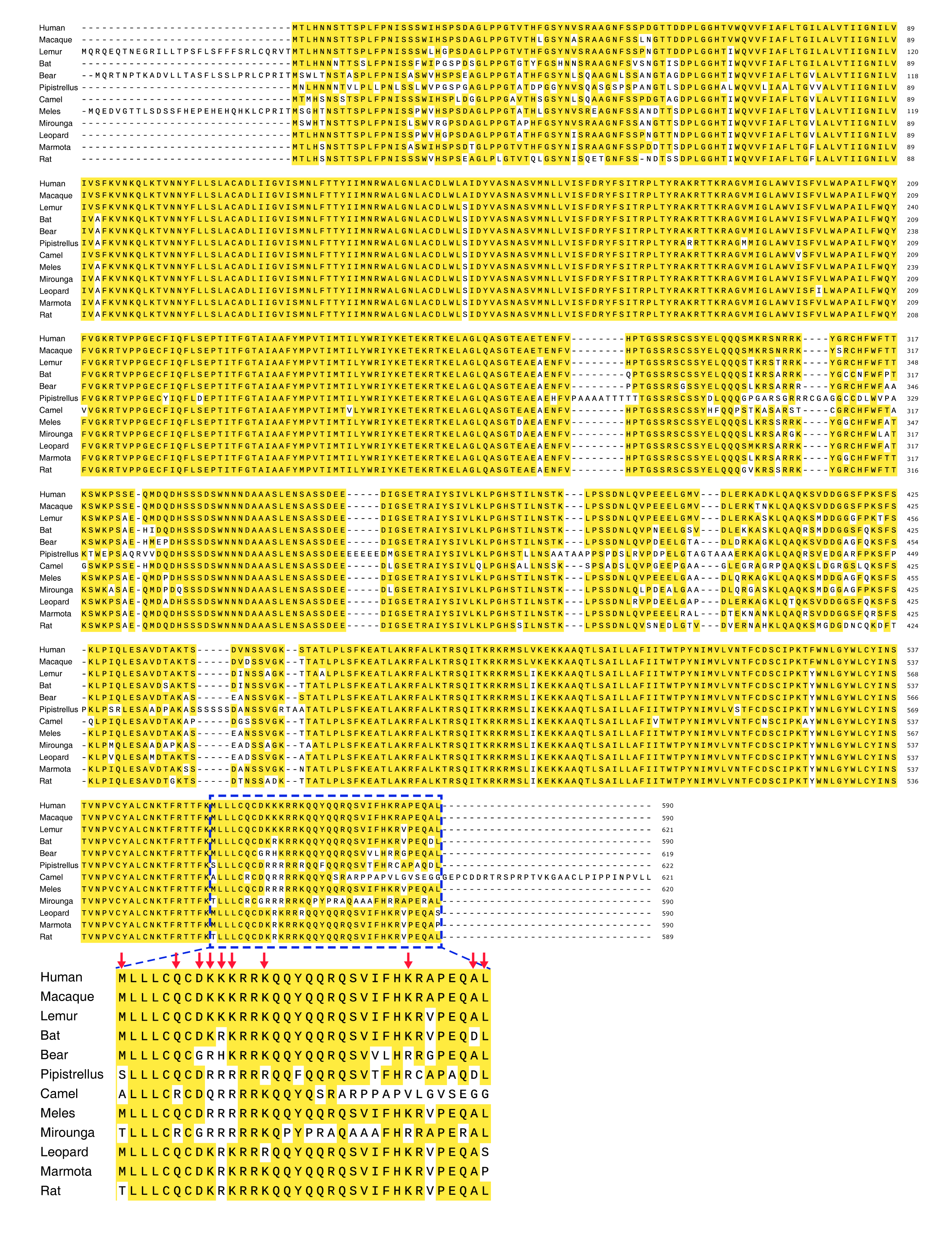

Supplement: SUPPLEMENTARY FIGURE S2 — Amino acid sequence alignments of M3R of humans, macaques, lemurs, bats, bears, pipistrellus, camels, meles, mirounga, leopards, marmotas, and rats. The bottom panel is an enlarged image showing the point mutations of hM3Ds-M1-M10 (red arrowhead). [file Image_2.TIF]

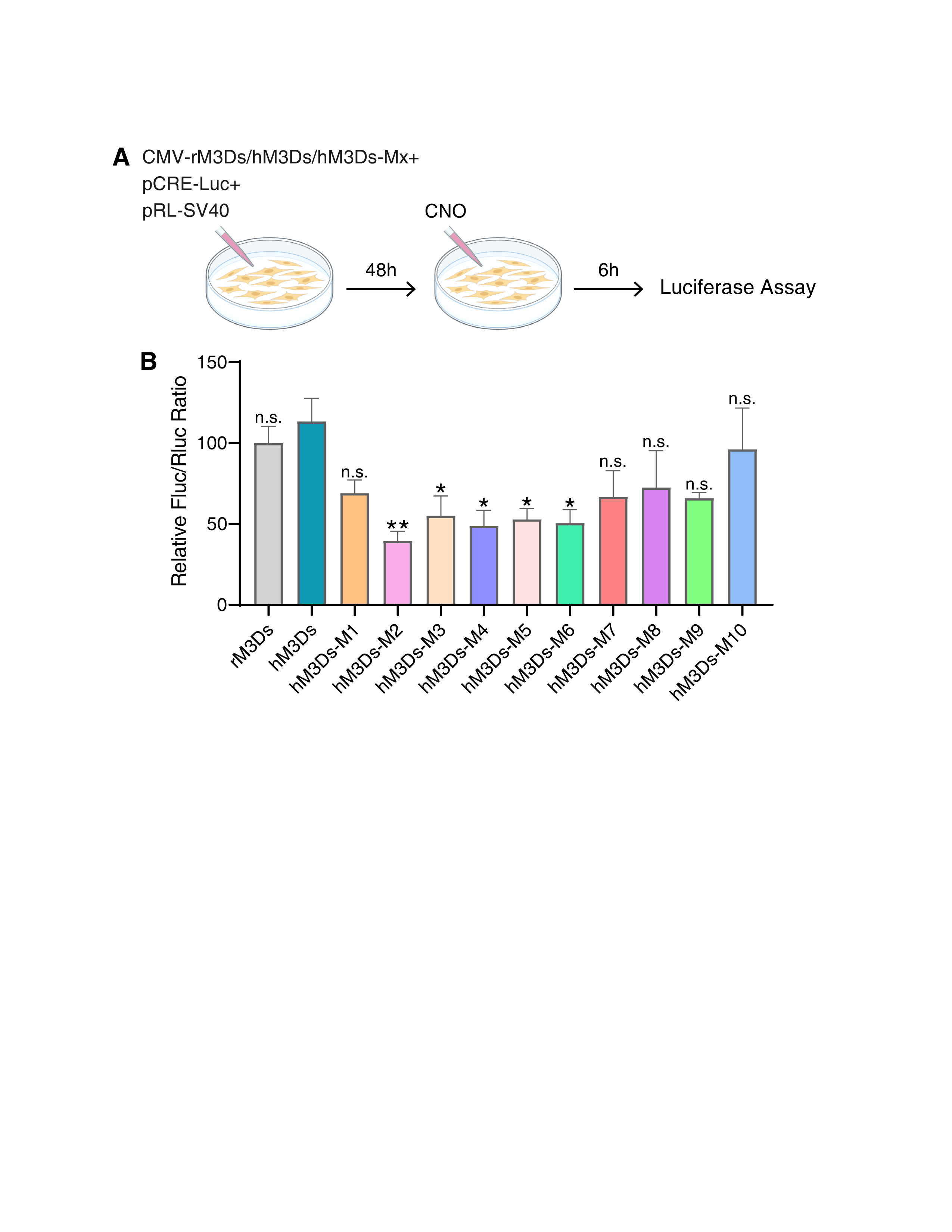

Supplement: SUPPLEMENTARY FIGURE S3 — Response of engineered hM3Ds mutants to CNO. (A) Experimental diagram showing assessment of the response of hM3Ds mutants to CNO, compared with rM3Ds and hM3Ds. (B) Relative luminescence fold changes elicited by rM3Ds, hM3Ds and hM3Ds mutants with excessive concentration of CNO at 10 μM. Data are normalized to the fold change elicited by hM3Ds and shown as mean ± SEM of triplicate experiments, one-way ANOVA with Dunnett’s post-hoc test, * p < 0.05, ** p < 0.01, n.s., not significant. [file Image_3.tif]
